# Application and evaluation of a rapid detection method based on two-dimensional PCR technology for hypervirulent *Klebsiella pneumoniae*

**DOI:** 10.3389/fmicb.2025.1554660

**Published:** 2025-06-19

**Authors:** Wenwen Zhu, Yiting Wang, Xin Jiang, Yan Zhao

**Affiliations:** Department of Laboratory Medicine, Jinshan Hospital, Shanghai Medical College, Fudan University, Shanghai, China

**Keywords:** 2D-PCR, hypervirulent *Klebsiella pneumoniae*, virulence genes, diagnosis, biomarkers

## Abstract

**Objectives:**

Hypervirulent *K. pneumoniae* (hvKp) is an emerging pathogen that is more virulent than classical *K. pneumoniae* (cKp). This study aimed to develop an economical, high-throughput, and accurate two-dimensional polymerase chain reaction (2D-PCR) assay for the rapid detection of hvKp.

**Materials and methods:**

Recombinant plasmids containing the *iucA*, *peg-344*, *rmpA2*, and *rmpA* virulence genes were constructed and used for assessing the sensitivity and specificity of the 2D-PCR. Clinical samples (*n* = 105) were collected and evaluated the performance of the 2D-PCR to comparison with conventional PCR methods.

**Results:**

The minimum detection limit of the 2D-PCR assay for *iucA*, *peg-344*, *rmpA2*, and *rmpA* were 10^3^, 10^2^, 10^3^, and 10^3^ copies/μL, respectively. Additionally, the concordance rates between the 2D-PCR and conventional PCR for detecting *iucA*, *peg-344*, *rmpA2*, and *rmpA* were all over 95%. The analysis revealed a sensitivity of 100.0% and a specificity of 96.2% when compared to conventional PCR.

**Conclusion:**

A 2D-PCR-based multiplex method for virulence genes of hvKp was successfully developed, demonstrating its outstanding features of high specificity, high sensitivity, and high throughput capability. This method could be used for the rapid diagnosis of infectious diseases caused by hvKp in clinical settings.

## Introduction

*Klebsiella pneumoniae* (*K. pneumoniae*) is one of the most common gram-negative opportunistic pathogens that are responsible for a variety of infectious diseases, including urinary tract infections, bacteremia, pneumonia, and liver abscesses (Wang et al., 2020; Stojowska-Swędrzyńska et al., 2021). At present, *K. pneumoniae* can be divided into the following two main types: classical *K. pneumoniae* (cKp) and hypervirulent *K. pneumoniae* (hvKp) (Zhang et al., 2023). cKp strains are frequently correlated with nosocomial infections or infections in a long-term care settings, indicating that a degree of immunocompromised state is essential for inducing disease by cKp strains (Walker and Miller, 2020). hvKp differs from cKp in its clinical and phenotypic characteristics. Patients infected with hvKp, who are typically younger and immunocompetent, present with more severe disease compared to those with cKp and originate from community settings (Choby et al., 2019). While the association of hvKp with community-acquired disease is more common (Pomakova et al., 2011), there has been an increasing number of reports suggesting the participation of hvKp isolates in healthcare-associated disease, particularly in pulmonary, ventilator, and healthcare-associated bacteremia (Zhu et al., 2021). An outbreak of carbapenem-resistant hvKp (CR-hvKp) in a hospital in China resulted in the death of five patients infected with ventilator-associated pneumonia (Gu et al., 2018). The increased transmission of hvKp strains in hospitals may raise the overall burden of this pathogen.

Currently, there is no consensus on the definition of hvKp, the accurate diagnosis and rapid identification of hvKp trains are essential for appropriate infection control measures. Traditional detection technologies, such as string tests, the mouse killing assay, and the *Galleria mellonella* infection model, cannot meet the fast and specific needs of clinical detection (Mai et al., 2023). Polymerase Chain Reaction (PCR) is an important detection technique in molecular biology, significantly enhancing the efficiency of pathogen detection (Schrader et al., 2012). Molecular diagnostic techniques, including traditional PCR, quantitative real-time polymerase chain reaction (qPCR), and other PCR assays, have been developed to identify hvKp and have shown satisfactory results. However, these methods are limited by low detection throughput and can cause nonspecific amplification (Schrader et al., 2012; Janik-Karpinska et al., 2022; Mai et al., 2023). A defining characteristic of hvKp strains is the presence of large and highly similar virulence plasmids pK2044 (224,152 bp) and pLVPK (219,385 bp), which harbor several virulence-encoding genes that confer the hypervirulent phenotype to hvKp (Chen et al., 2004; Wu et al., 2009). Therefore, the virulence genes on plasmids can be used as specific hypervirulence markers for the clinical detection of hvKp, such as *rmpA* and *rmpA2* (capsular polysaccharide (CPS)-regulating genes), *peg-344* (metabolism genes), and *iucA* (siderophore genes).

Two-dimensional polymerase chain reaction (2D-PCR) is a multiplex PCR detection method that identifies multiple target genes in the same PCR tube using base-quenched probe technology and fluorescence melting temperature (Mao et al., 2018; Zhan et al., 2020). In 2D-PCR systems, a synthesized pre-tag sequence located at the 5′ end of a specific primer is utilized, and some bases in the pre-tag sequence are changed, allowing one probe to recognize multiple tags with various melting temperature (Tm) values. Therefore, this study aimed to establish a rapid molecular diagnostic method to accurately identify hvKp by detecting the virulence genes *iucA*, *peg-344*, *rmpA2*, and *rmpA*.

## Materials and methods

A total of 105 nonrepetitive *K. pneumoniae* strains were collected from the clinical laboratory of Fudan University Affiliated Jinshan Hospital from January to June 2024, these isolates were identificated using AUTOF MS 1000 AUTOBIO (Autobio Diagnostics, Zhengzhou, China). *K. pneumoniae* strain RJF293 (Xu et al., 2021) and *K. pneumoniae* strain HS11286 (Liu et al., 2012) were used as positive control and negative control, respectively. The nucleotide sequences of *rmpA* (633 bp), *rmpA2* (637 bp), *iucA* (1725 bp), and *peg-344* (903 bp) from the virulence plasmid pRJF293 of *K. pneumoniae* RJF293 were cloned into the pMD20-T vector (TaKaRa, Japan). The positive recombinant plasmid was subsequently transferred into *Escherichia coli DH5α* (Tiangen Biotech, Beijing, China) and confirmed by sequencing. The gene sequences of *iucA*, *peg-344*, *rmpA2*, and *rmpA* were downloaded from the GenBank database. Subsequently, the primers were designed using Primer Premier 5 software. The sequences of the primers and probes are shown in Table 1.

**Table 1:** Primers and universal probe used in this study.

Primer	Sequences (5′ → 3′)	Amplicons size (bp)	References
2D-PCR			
peg-344-F2	CCATTACC**TTG**CTTATACACTTCCACAGCGAAAGAATAACCCCAG	160	Zhu et al. (2022) This study
Peg-344-R2	GGAAAGGACAGAAAGCCAG		
p-rmpA-F2	CCATTACCAACCTTATACACTTCCACTTCAGGGAAATGGGGAGGGTA	252	Zhu et al. (2022) This study
p-rmpA-R2	CATTGCAGCACTGCTTGTTCC		
p-rmpA2-F2	CCATTACC**T**ACCTTATACACTTCCACGTTAACTGGACTACCTCTGGTTT	104	Wu et al. (2022) This study
p-rmpA2-R2	ATCCGGCTATCAACCAATACTC		
iucA-F2	CCATTACC**T**A**G**CTTATACA**T**TTCCACTGTTTACGGCTGAAGCGGAT	101	Zhu et al. (2022) This study
iucA-R2	CACGGTAGATAAGCCCGACC		
intI-P	FAM-CCATTACCAACCTTATACACTTCCAC-P		Zhu et al. (2022)
Traditional PCR			
Peg344-F	CTTGAAACTATCCCTCCAGTC	508	Luo et al. (2023)
Peg344-R	CCAGCGAAAGAATAACCCC		
rmpA-F	TTAACTGGACTACCTCTGTTTCAT	332	Luo et al. (2023)
rmpA-R	AATCCTGCTGTCAACCAATACT		
rmpA2-F	ATCCTCAAGGGTGTGATTATGAC	430	Luo et al. (2023)
rmpA2-R	CCTGGAGAGTAAGCATTGTAGAAT		
iucA-F	CTCTTCCCGCTCGCTATACT	239	Luo et al. (2023)
iucA-R	GCATTCCACGCT TCACTTCT		

The primer tags were underlined, and point mutations within these tags were highlighted in bold.

### DNA extraction

The genomic DNA of *K. pneumoniae* was obtained using the Tianamp Bacteria DNA Kit (Tiangen Biotech, Beijing, China) according to the manufacturer’s instructions. Plasmid DNA of *E. coli DH5α* was obtained by using the TianampTIANprep Mini Plasmid Kit (Tiangen Biotech, Beijing, China). Finally, approximately 80 μL of the DNA solution was prepared to serve as a template for the DNA reaction. The extracted DNA concentration was measured with a NanoDrop™ 2000 spectrophotometer (NanoDrop Technologies, LLC, Wilmington, DE, USA) and stored at −20°C for subsequent analysis.

### Traditional PCR assays for biomarkers

The virulence-associated factors *iucA*, *rmpA*, *rmpA2*, and *peg-344* were investigated using the traditional PCR method. For each reaction, 12.5 μL Premix Taq (TaKaRa, Japan), 0.5 μL of each primer (10 μM), 1 μL genomic DNA (approximately 100 ng/μL) and 10.5 μL of deionized water (HPLC grade). PCR was performed under the following cycling conditions: 94°C for 4 min, followed by 35 cycles of 94°C for 30 s, 55°C for 30 s and 72°C for 30 s, with a final extension at 72°C for 10 min, these reactions were conducted in a thermal cycler (T-100, BioRad, USA). Each run included a positive control (*K. pneumoniae* RJF293), a negative control (*K. pneumoniae* HS11286) and a blank control (deionized water). The PCR products were analyzed by electrophoresis on a 0.8% agarose gel and visualized with UV light.

### 2D-PCR

The 2D-PCR assays were run on the QuantStudio 3 (Thermo Fisher, USA), with each reaction containing a total volume of 25 μL. The FAM channel was employed for all experiments. The reaction mixture contained 2.5 μL 10 × PCR buffer (Mg^2+^ free), 1.5 μL MgCl_2_ (25 mM), 0.7 μL dNTP Mixture (2.5 mM each), 0.5 μL Taq Hot Start Polymerase (TaKaRa, Japan), 0.1 μL each of labeled forward primer, 0.6 μL reverse primer, 0.4 μL FAM probe, 2 μL extracted DNA template, the volume was adjusted to 25 μL with nuclease-free water. The 2D-PCR cycling parameters were as follows: an initial heating step at 95°C for 10 min, followed by 40 cycles of 95°C for 10 s, and 60°C for 30 s. Following the amplification, a melting curve analysis was performed, starting at 30°C for 4 min and gradually increasing by 0.1 C/s until reaching 80°C. The fluorescence signal was acquired every second during this step. Finally, a cooling step was carried out at 40°C for 30 s.

## Results

### 2D-PCR specificity for identifying hvKp

**Figure 1:**
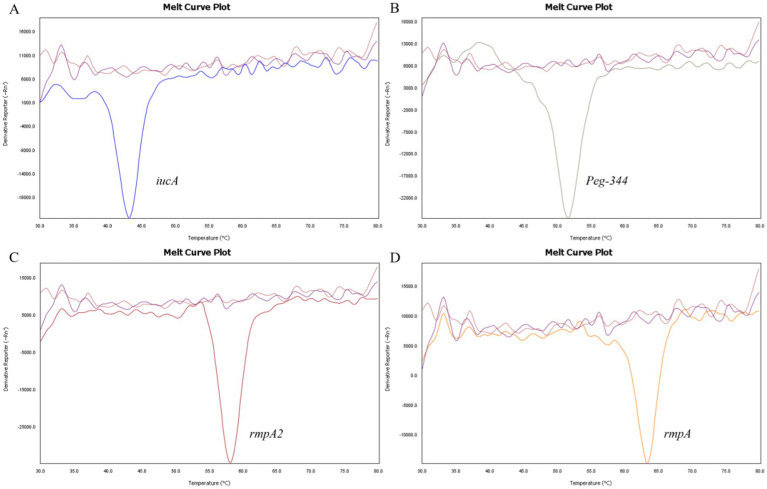
Single gene detection by 2D-PCR. **(A)** *iucA*, **(B)** *peg-344*, **(C)** *rmpA2*, and **(D)** *rmpA*. The approximate Tm values corresponding to *iucA*, *peg-344*, *rmpA2*, and *rmpA* are 43°C, 52°C, 58°C, and 64°C, respectively. The curves without dissolution peaks represent the negative control and the blank control, respectively.

**Figure 2:**
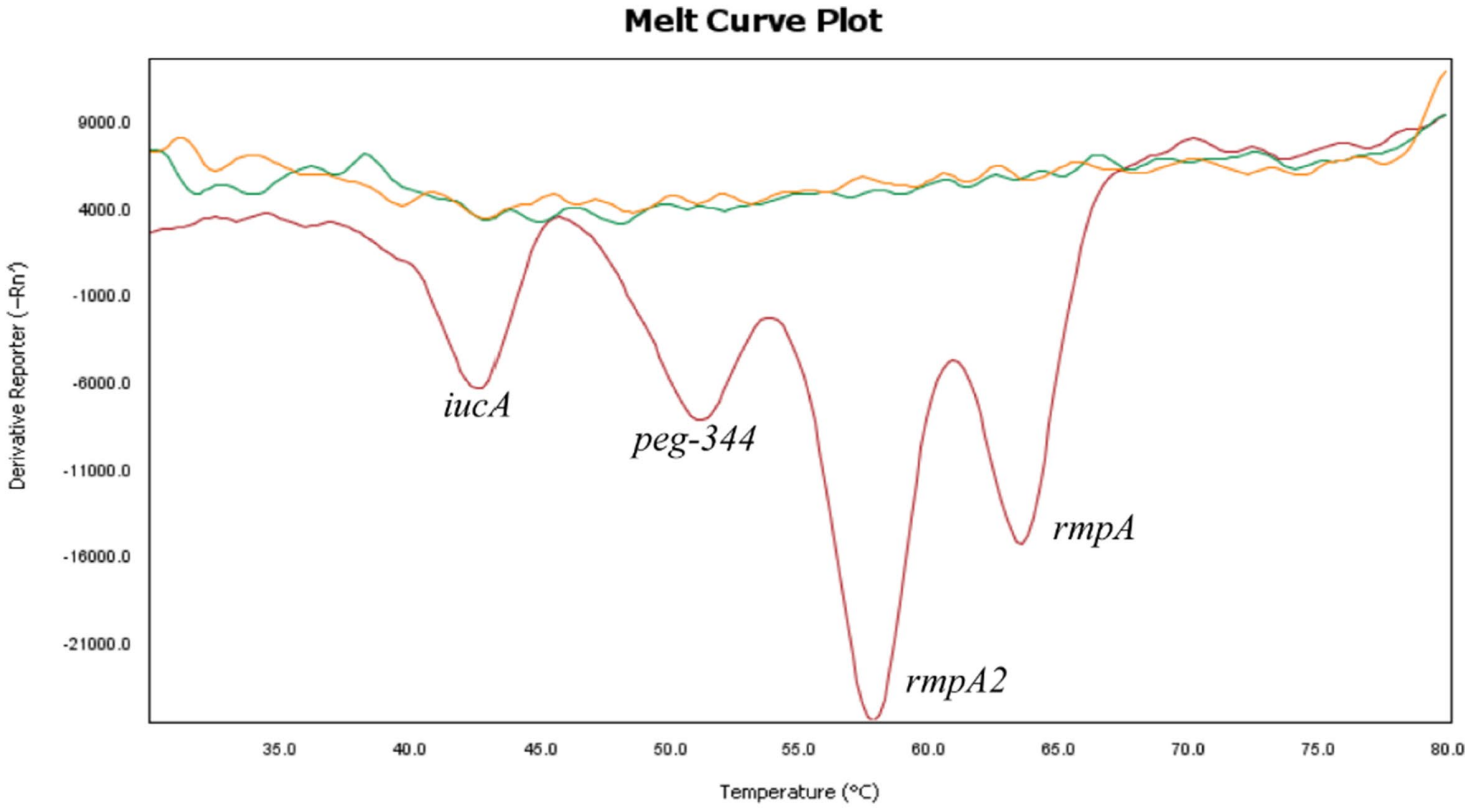
The presence of four melting valleys in the mixture of four gene plasmids confirmed the simultaneous detection of all four target genes. The curves without dissolution peaks represent the negative control and the blank control, respectively.

The specificity of the 2D-PCR test to detect *iucA*, *peg-344*, *rmpA2,* and *rmpA* was evaluated using positive plasmid samples with concentrations of approximately 10^5^ copies/μL. First, a single positive plasmid was used as template to confirm the specificity of the primers. The results demonstrate that the melting curves are clear, with the Tm of *iucA*, *peg-344*, *rmpA2*, and *rmpA* being approximately 43°C, 52°C, 58°C, and 64°C, respectively (Figure 1). The Tm difference between the four target genes is at least 5°C, which makes them easy to distinguish. To assess the capability of 2D-PCR in simultaneously detecting multiple target genes, a mixture of positive plasmids containing four virulence genes was utilized as a DNA template. The results showed that 2D-PCR successfully detected all four genes simultaneously. As shown in Figure 2, the 2D-PCR technique effectively detected four virulence genes simultaneously, with clearly distinguishable peaks for each target. Thus, this assay was highly specific for the detection of *iucA*, *peg-344*, *rmpA2*, and *rmpA* from hvKp.

### 2D-PCR sensitivity for identifying hvKp

**Figure 3:**
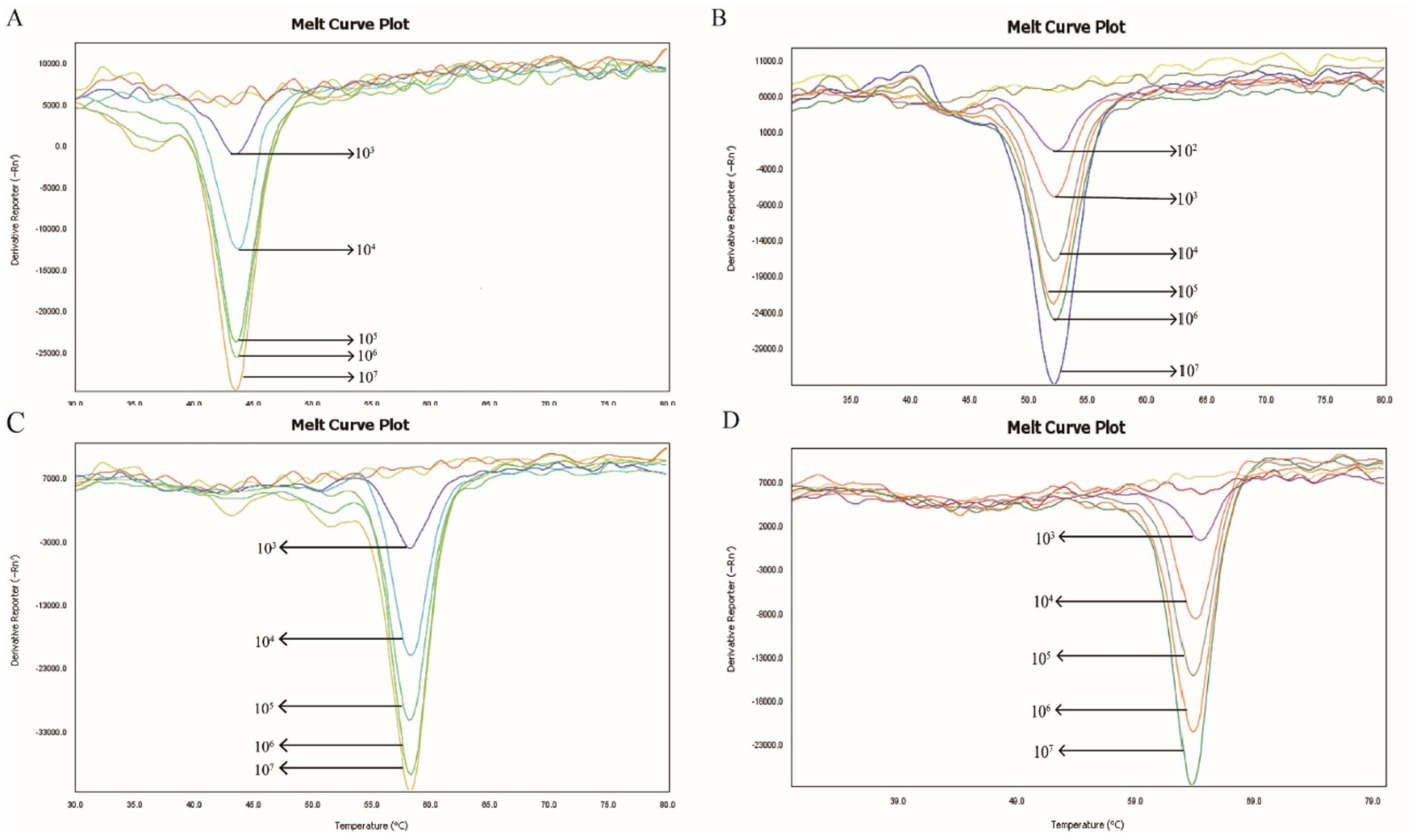
Sensitivity test of 2D-PCR. The minimum detectable concentration of **(A)** *iucA*, **(B)** *peg-344*, **(C)** *rmpA2* and **(D)** *rmpA* were 10^3^, 10^2^, 10^3^, and 10^3^ copies/μL, respectively. The curves without dissolution peaks represent the negative control and the blank control, respectively.

To assess the minimum detection limit of the 2D-PCR system, four virulence plasmid samples were serially diluted across a gradient ranging from 10^7^–10^1^ copies/μL. As shown in Figure 3, all corresponding targets were successfully detected in samples with a concentration of 10^3^ copies/μL. However, only the *peg-344* target gene was detected in samples at 10^2^ copies/μL, and no corresponding targets were identified in samples below this concentration. The lowest detectable concentrations of this method for the *iucA*, *peg-344*, *rmpA2*, and *rmpA* genes were 10^3^, 10^2^, 10^3^, and 10^3^ copies/μL, respectively. Meanwhile, the signal for each target gradually weakened as the template concentration decreased.

### Testing of clinical samples

**Figure 4:**
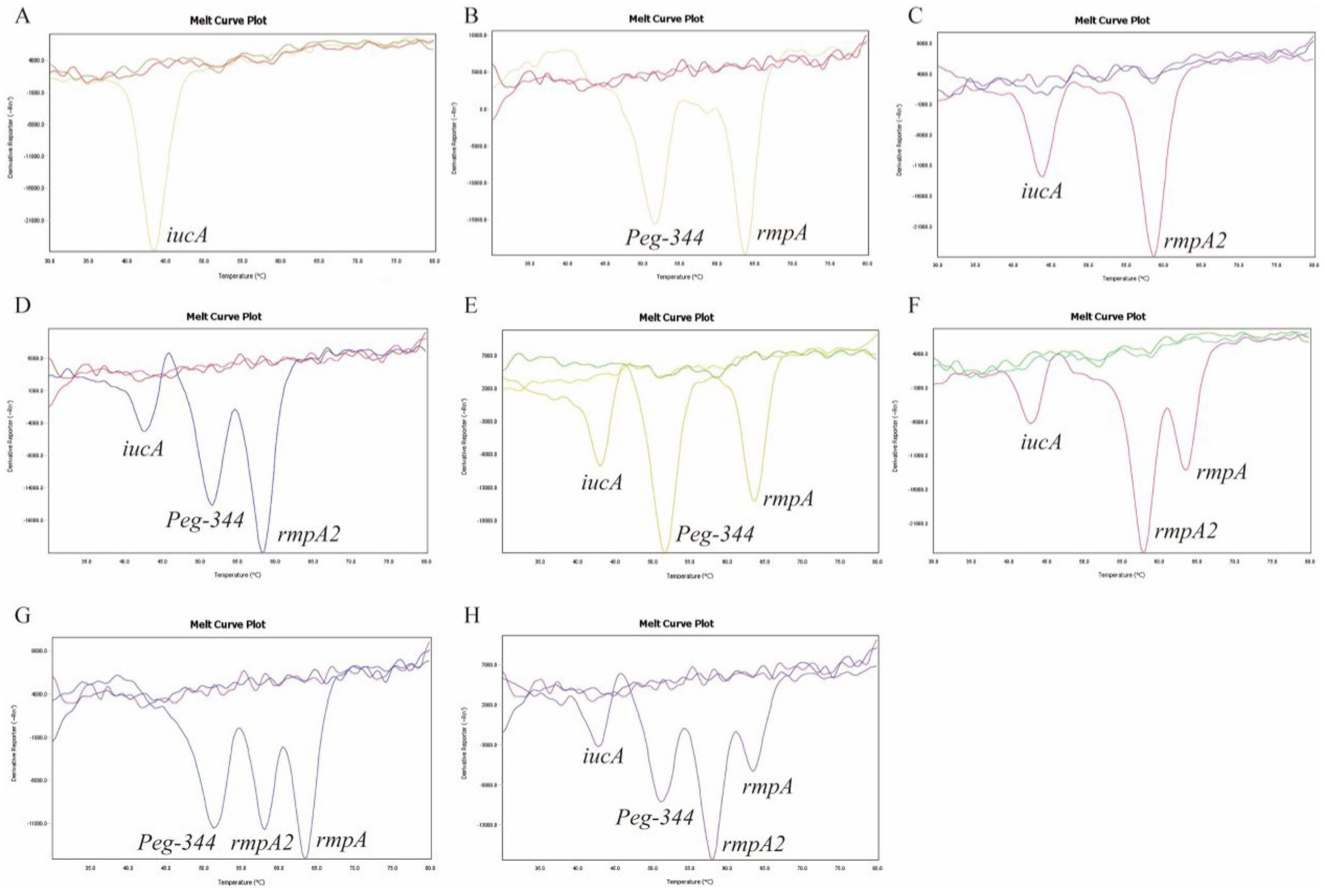
Clinical samples were examined to identify hvKp strains using 2D-PCR. The curves without dissolution peaks represent the negative control and the blank control, respectively.

**Figure 5:**
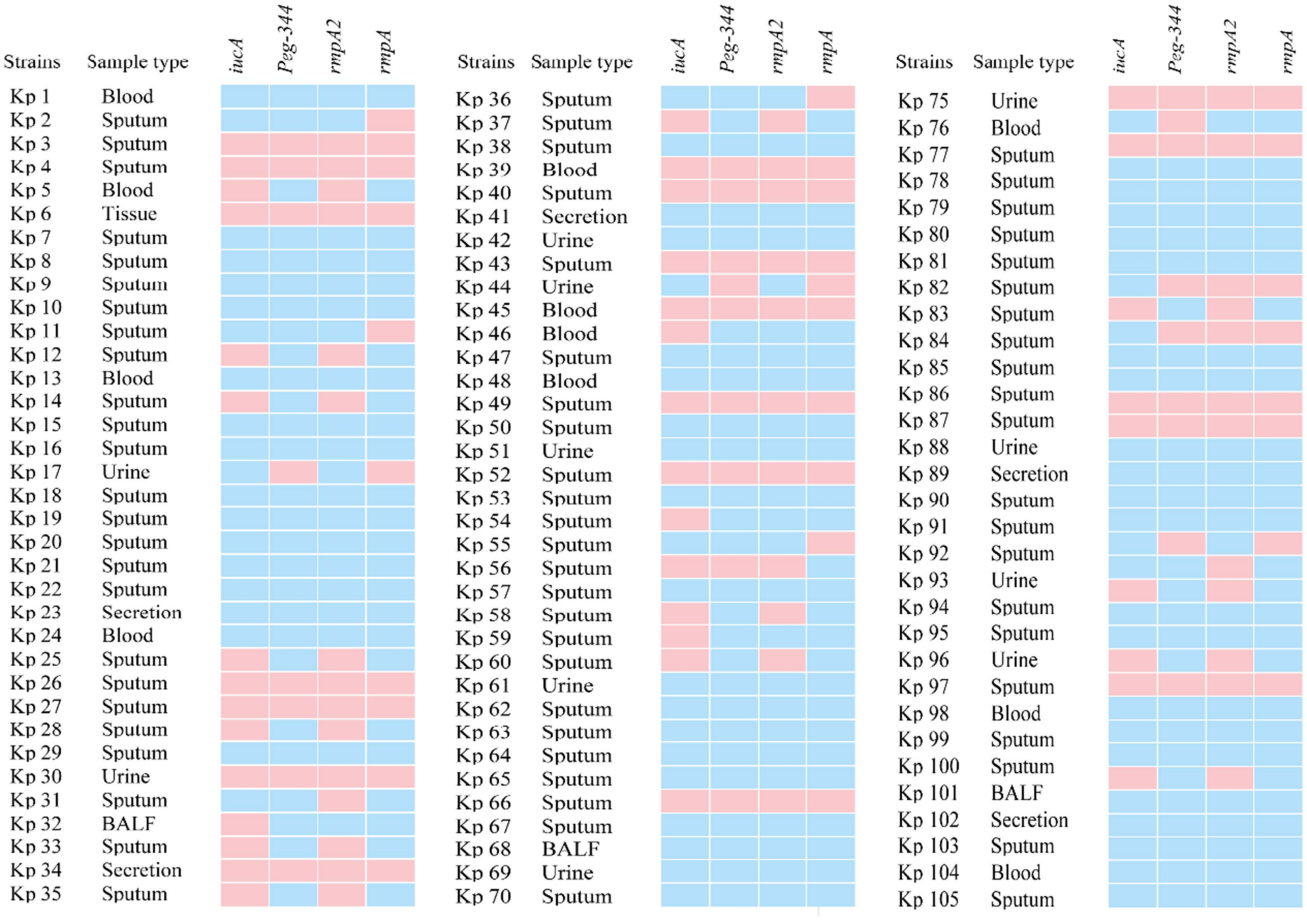
The heat map of virulence genes in 105 *K. pneumoniae* isolates showed the prevalence of these genes across different clinical samples, with blue indicating the presence of a virulence gene and pink representing its absence. The strain in which four virulence genes, *iucA*, *peg-344*, *rmpA2* and *rmpA*, were simultaneously present was identified as hvKp. BALF: Bronchoalveolar lavage fluid.

All 105 samples confirmed strains of *K. pneumoniae* were tested using the proposed 2D-PCR system (Figure 4). In 105 samples, 2D-PCR detected 55 samples of hvKp positive (*iucA*, *peg-344*, *rmpA2*, and *rmpA*) and 50 samples of other types that were negative (Figure 5). The positive rate of *iucA*, *peg-344*, *rmpA2*, and *rmpA* were 63.8, 75.2, 63.8, and 73.3%, respectively. There are differences in the ability of 2D-PCR and conventional PCR to detect the four virulence genes, for the detection of *iucA* and *peg-344*, the concordance between both methods was 99.1%. Among these, two samples tested positive in 2D-PCR and negative in conventional PCR. For the detection of *rmpA2*, the concordance rate is 97.1%, with three samples showing positive results in 2D-PCR but negative in conventional PCR. The results for *rmpA* detection were completely consistent between the two methods. All samples that tested positive by 2D-PCR also tested positive in conventional PCR, while all inconsistent results were positive in 2D-PCR and negative in conventional PCR (Table 2). Compared with traditional PCR, the specificity of 2D-PCR is 96.2%, and the sensitivity is 100.0%.

**Table 2:** Comparison of 2D-PCR and conventional PCR results.

Virulence genes	2D-PCR	Conventional PCR		Total samples	Concordance rate (%)
		Positive samples	Negative samples		
*iucA*	Positive samples	66	1	67	99.1
	Negative samples	0	38	38	
*peg-344*	Positive samples	78	1	79	99.1
	Negative samples	0	26	26	
*rmpA2*	Positive samples	64	3	67	97.1
	Negative samples	0	38	38	
*rmpA*	Positive samples	77	0	77	100.0
	Negative samples	0	28	28	

## Discussion

HvKp is undergoing spread globally as a notorious clinical pathogen causing multiple severe infections. In the past, hvKp has been perceived as having a hypermucoviscosity phenotype, exhibiting susceptibility to antimicrobial agents, and a propensity to induce invasive infections among healthy individuals in the community (Han et al., 2024). However, partly due to antibiotic abuse, multidrug-resistant hvKp, particularly CR-hvKp, has garnered significant attention in recent years. CR-hvKp, which is associated with high morbidity and mortality rates, poses a significant challenge to infection control and clinical treatment (Pu et al., 2023). HvKp with high virulence and epidemic potential is threatening human health. Improving the identification of hvKp and achieving early and accurate detection is crucial to support subsequent effective treatment among patients.

A common feature of hvKp is the combined expression of multiple virulence factors that serve as reliable biomarkers for accurately differentiating hvKp from cKp. Previous studies have established a PCR detection method for hvKp based on the combination of *peg-344*, *iroB*, *iucA*, *_p_rmpA*, and *_p_rmpA2*, along with siderophore production (SP) exceeding 30 μg/mL, with accuracy greater than 95% (Russo et al., 2018). Cai et al. developed a multiplex q-PCR assay targeting *iroB*, *iucA*, *rmpA*, and *rmpA2* as molecular biomarkers for the rapid detection of hvKp. This assay demonstrated 100% sensitivity and 98% specificity in clinical validation studies (Cai et al., 2025). In this study, a 2D-PCR analysis of four virulence genes was conducted on 105 clinical isolates. The positive rates of the hvKP virulence genes were detected as *peg-344*, *rmpA*, *iucA*, and *rmpA2* in descending order, and the detection rate of four virulence genes were more than 60%. *Peg-344* is widely distributed on the virulence plasmid of hvKp strains, and appears to be hvKp-specific (Bulger et al., 2017). Liao et al. (2020) established a LAMP detection method for hvKp based on *peg-344* as a target molecular biomarker, with an accuracy of 97%, a sensitivity of 99% and a specificity of 96%. *rmpA* and *rmpA2* are regulatory genes for polysaccharide expression in the capsule of hvKp, which decrease capsule production and virulence of strains if missing (Matono et al., 2022). Yan et al. (2023) effectively developed a recombinase-aided amplifcation (RAA) detection method targeting *peg-344* and *rmpA*, enabling the rapid identification of hvKp in clinical samples with both 100% sensitivity and specificity. *The iucA* genes that are responsible for producing aerobactin and salmochelin also demonstrate specificity in hvKp (Mydy et al., 2020). These biomarkers have the potential to be used as a rapid diagnostic test for differentiating hvKp from cKp. Therefore, the four virulence genes *iucA*, *peg-344*, *rmpA2*, and *rmpA* in this study can be used as accurate molecular markers for hvKP (Table 3).

**Table 3:** Genes, detection time, specificity, sensitivity, accuracy, advantages, and disadvantages of hvKp detection methods based on biomarkers.

Method	Genes	Detection time	Specificity (%)	Sensitivity (%)	Accuracy (%)	Advantage	Disadvantage	References
PCR^1^	*peg-344*, *iroB*, *iucA*, *_p_rmpA*, and *_p_rmpA2*	1 h			95	Strong specificity	Low throughput requires post-amplification analysis and specialized instruments	Russo et al. (2018)
q-PCR^2^	*iroB, iucA, rmpA, and rmpA2*	1–2 h	98	100		High sensitivity and specificity	Low throughput	Kuypers et al. (2017); Cai et al. (2025)
LAMP^3^	*peg-344*	15-60 min	96	99		No specialized equipment required High sensitivity and specificity	Prone to non-specific amplification	Cai et al. (2025)
RAA^4^	*peg-344*, *rmpA*	10–20 min.	100	100		Rapid results without specialized equipment High sensitivity and specificity	The design requirements for primer probes are high	Yan et al. (2023)
2D-PCR^5^	*iucA, peg-344, rmpA, and rmpA2*	1 h	96. 2	100		High throughput High sensitivity and specificity	Primer probe labels design requirements are high	This study

1 Polymerase chain reaction.

2 Quantitative real-time polymerase chain reaction.

3 Loop-mediated isothermal amplification.

4 Recombinase-aided amplification.

5 Two-dimensional polymerase chain reaction technology.

Traditional PCR and multiplex PCR are often used to detect virulence genes (Compain et al., 2014; Albasha et al., 2020). The traditional PCR method has significant disadvantages in clinical diagnosis, such as long processing time, the need for electrophoresis after amplification to identify the results, and the limitation that a specific primer pair can only detect one gene type (Pan et al., 2025). Multiplex PCR has some limitations, such as false-positive results, a long processing cycle, complex workflows, and low efficiency (Dessajan and Timsit, 2024). Therefore, researchers have made significant efforts in recent years to improve and develop molecular methods for hvKp identification, such as RAA and LAMP. Compared with existing hvKp detection methods, 2D-PCR overcomes the inherent limitations of multiplex PCR by introducing Tm as the second dimension, which strictly relies on proportional relationships among fluorescence channels, probes, and detectable targets. A high-throughput closed-tube detection system has been established, significantly improving the detection throughput of single-tube reactions and reducing false positives caused by nonspecific amplification (Wang et al., 2025). Its probe design strategy allows a single probe to recognize multiple targets, greatly reducing the number of probes required. Compared with conventional probes, 2D-PCR probes do not require quenchers, significantly reducing detection costs. The experimental process is simplified, eliminating the need for subsequent operations such as electrophoresis, making it suitable for large-scale clinical screening.

2D-PCR is a closed-tube multiplex PCR technology that combines melting curve analysis with PCR and is simple and sensitive for clinical applications. 2D-PCR is considered an excellent amplification detection technology widely used for the quantification of various pathogens, assisting in the diagnosis of various diseases such as human papillomavirus (HPV) (Wu et al., 2022) and inflammatory bowel disease (Wu et al., 2024). In the present study, a 2D-PCR method was established for the detection of hvKp. To successfully detect multiple genes in a single tube while reducing the number of probes required for the detection of multiple genes, we introduced pre-labeled sequences and probes that can recognize multiple target genes in the 2D-PCR reaction system and evaluated the sensitivity and specificity of this method. The experimental results indicate that the melting temperature difference of the four virulence genes was about 5°C, and there was no cross-reactivity. When a mixture of four virulence plasmids is used as a template, 2D-PCR can detect four virulence genes in a single closed-tube assay. Simultaneously, we found that the minimum detection limit of *iucA*, *peg-344*, *rmpA2*, and *rmpA* genes were 10^3^, 10^2^, 10^3^, and 10^3^ copies/μL, respectively. The limit of detection in this study was consistent with the multiplex q-PCR method for detecting hvKP reported by Cai et al. (2025). Finally, to evaluate the clinical applicability of our 2D-PCR assay, we screened 105 clinical samples using 2D-PCR and conventional PCR. The sensitivity and specificity of the 2D-PCR assay were 100 and 96.2% compared with conventional PCR as the reference standard. The 2D-PCR method developed in this study demonstrates a high degree of concordance with traditional PCR results in clinical practice. This method can be effectively employed for the detection of clinical samples, providing a rapid diagnosis of hvKp in clinical samples.

The definition of hvKp virulence genes remains controversial, and further research and verification are necessary to determine which virulence genes should be fully defined as hvKp (Kocsis, 2023). So, this study has some limitations. First, the selection of virulence genes is not comprehensive enough. We only detected four virulence genes, this can be addressed by increasing the number of fluorescent channels and tag sequences to improve the detection throughput of 2D-PCR, it is highly possible for 2D-PCR to identify more than 30 genes simultaneously. Second, the number of clinical samples we selected is relatively small, and further expansion of the sample size to evaluate the clinical application potential of 2D-PCR comprehensively.

## Conclusion

This study presents a simple, high-throughput method for the rapid identification of biomarkers for hvKp strains, enabling accurate detection of hvKp with excellent specificity and sensitivity. Additionally, 2D-PCR also shows high specificity in clinical performance analyses, meeting the needs of clinicians diagnosing hvKp. Thus, 2D-PCR is a powerful tool for early diagnosis and epidemiological surveillance of hvKp.

## Data Availability

The original contributions presented in the study are included in the article/supplementary material, further inquiries can be directed to the corresponding author.
